# Effect of meloxicam treatment on movement asymmetry in riding horses in training

**DOI:** 10.1371/journal.pone.0221117

**Published:** 2019-08-13

**Authors:** Emma Persson-Sjodin, Elin Hernlund, Thilo Pfau, Pia Haubro Andersen, Karin Holm Forsström, Marie Rhodin

**Affiliations:** 1 Department of Anatomy, Physiology and Biochemistry, Swedish University of Agricultural Sciences, Uppsala, Sweden; 2 Department of Clinical Science and Services, The Royal Veterinary College, North Mymms, Hatfield, United Kingdom; 3 Department of Clinical Sciences, Swedish University of Agricultural Sciences, Uppsala, Sweden; 4 Equine Clinic, University Animal Hospital, Uppsala, Sweden; University College Dublin, School of Veterinary Medicine, IRELAND

## Abstract

Quantitative gait analysis has revealed that a large proportion of horses in training, perceived as free from lameness by their owners, show movement asymmetries of equal magnitude to horses with mild clinical lameness. Whether these movement asymmetries are related to orthopaedic pain and/or pathology has yet to be further investigated. Therefore, the objective of this study was to determine whether movement asymmetries in riding horses in training are affected by anti-inflammatory treatment with meloxicam. In a crossover design, horses were treated with meloxicam or placebo for four days respectively, with a 14–16 day washout period between treatments. Objective movement analysis utilising body mounted accelerometers was performed on a hard and a soft surface before and on day four of each treatment. A trial mean was calculated for the differences between the two vertical displacement minima and maxima of head (HDmin, HDmax) and pelvis (PDmin, PDmax) per stride. Horses (n = 66) with trial mean asymmetries greater than 6 mm for HDmin or HDmax, or more than 3 mm for PDmin or PDmax, at baseline were included. The difference before and after each treatment in the measured movement asymmetry was assessed with linear mixed models. Treatment with meloxicam did not significantly affect the movement asymmetry in any of the models applied (all p>0.30). These results raise new questions: are the movement asymmetries in riding horses in training simply expressions of biological variation or are they related to pain/dysfunction that is non-responsive to meloxicam treatment?

## Introduction

Disorders of the locomotor apparatus are collectively the most common reason for equine veterinary consultation [1,2]. They are also the most common cause of euthanasia in the Swedish riding horse population [3].

A major shortcoming of subjective visual assessment of lameness is the low inter-observer agreement [4–7], probably in part due to limitations of the human visual perception of asymmetry [8,9]. Several movement symmetry based objective systems have been developed (for a review see Serra Bragança, Rhodin and van Weeren [10]) with the aim of obtaining a more accurate detection of lameness. Studies indicate that these systems might be more sensitive for detection of low grade lameness when compared to subjective lameness evaluation by experienced observers [11–13].

However, the currently recommended thresholds [14] for one of these movement symmetry based objective systems frequently result in a classification of horses in training and perceived as free from lameness by their owner, as asymmetric. In a prevalence study of 222 riding horses in training, 73% had movement asymmetries [15] with median asymmetry values for both forelimbs and hind limbs in the same range as in horses with clinically relevant movement deficits measured with the same objective system [16,17]. Other studies detected asymmetries exceeding the aforementioned thresholds in 47% of 201 [18], or values outside normal ranges according to Buchner *et al*. [19] in 67% of 27 [20] riding horses in training. Using subjective lameness assessment 53% of 57 [21] and 38% of 506 [22] horses in regular work were classified as lame, suggesting that this finding is also inherent to subjective scoring systems.

It is currently unclear to what extent movement asymmetries are related to pain and/or pathology or whether they may represent natural biological variation in the movement patterns of these horses. Thus, the relation between clinically significant movement asymmetry and biological variation of movement symmetry needs to be investigated further. It is of particular interest to investigate methods for reliable discrimination between asymmetry and lameness as early detection of orthopaedic diseases is integral to avoid progression into chronic states of disease that carry a poor prognosis for recovery in spite of treatment. If movement asymmetries in horses in training are decreased by treatment with a nonsteroidal anti-inflammatory drug, it would be reasonable to assume that such asymmetries are due to pain from an underlying unrecognized inflammatory process.

Meloxicam is a nonsteroidal anti-inflammatory drug (NSAID) of the oxicam class and acts by inhibition of prostaglandin synthesis via preferential COX-2 (inducible cyclooxygenase isoform) inhibition [23]. Oral suspension of meloxicam is approved in the European Union since 1998 [24] for the treatment of acute and chronic locomotive disorders in equines and is commonly used in many European countries with this indication. In horses with experimentally induced lameness from lipopolysaccharide-induced joint inflammation, meloxicam treatment has been shown to result in a reduction in visual lameness scores [25–27], a reduction in head movement asymmetry [27] and in a suppression of synovial fluid markers of inflammation, MMP activity and cartilage turnover [25] when compared to placebo. In horses with clinical musculoskeletal pain, meloxicam was slightly more effective in alleviating lameness than vedaprofen [28]. Collective evidence thus suggests that meloxicam reduces musculoskeletal pain of inflammatory origin.

The aim of this study was to determine whether movement asymmetries in riding horses, in training and perceived as free from lameness by their owner, are affected by anti-inflammatory treatment with meloxicam. The hypothesis was that, if a proportion of the horses selected for the study suffered from musculoskeletal pain of inflammatory origin, meloxicam treatment would decrease the degree of movement asymmetry on a group level.  

## Materials and methods

The study protocol was approved by the Ethical Committee for Animal Experiments, Uppsala, Sweden, application number C 48/13 and C 92/15. Informed written consent was obtained from all horse owners. Data collection was performed during the years 2013–2016.

### Horses

Convenience sampled warmblood riding horses (privately owned, horses belonging to two equestrian centres and two riding schools) stabled in the vicinity of the University of Agricultural Sciences in Uppsala were screened for movement asymmetry using a commercially available inertial measurement unit (IMU) sensor system (Lameness Locator, Equinosis, Columbia, MO, USA). Screened horses were included based on the following criteria: 1. they were in full training; 2. they were considered free from lameness by their owner; 3. they had not been treated for lameness during the two months preceding data collection. Horses presenting with asymmetry values above thresholds previously used in a prevalence study [15] for head (absolute value >6 mm) or pelvic (absolute value >3 mm) parameters were included in the study. The values were obtained from measurements on hard and/or soft surface, at the preferred speed of each horse trotted in-hand on the straight. Horses considered too lame (>2 degrees on a 0–5 ordinal scale) to continue their normal training at inclusion or at a later time point during the study, were excluded from the study, at the discretion of the study veterinarians.

### Study design

**Fig 1:**
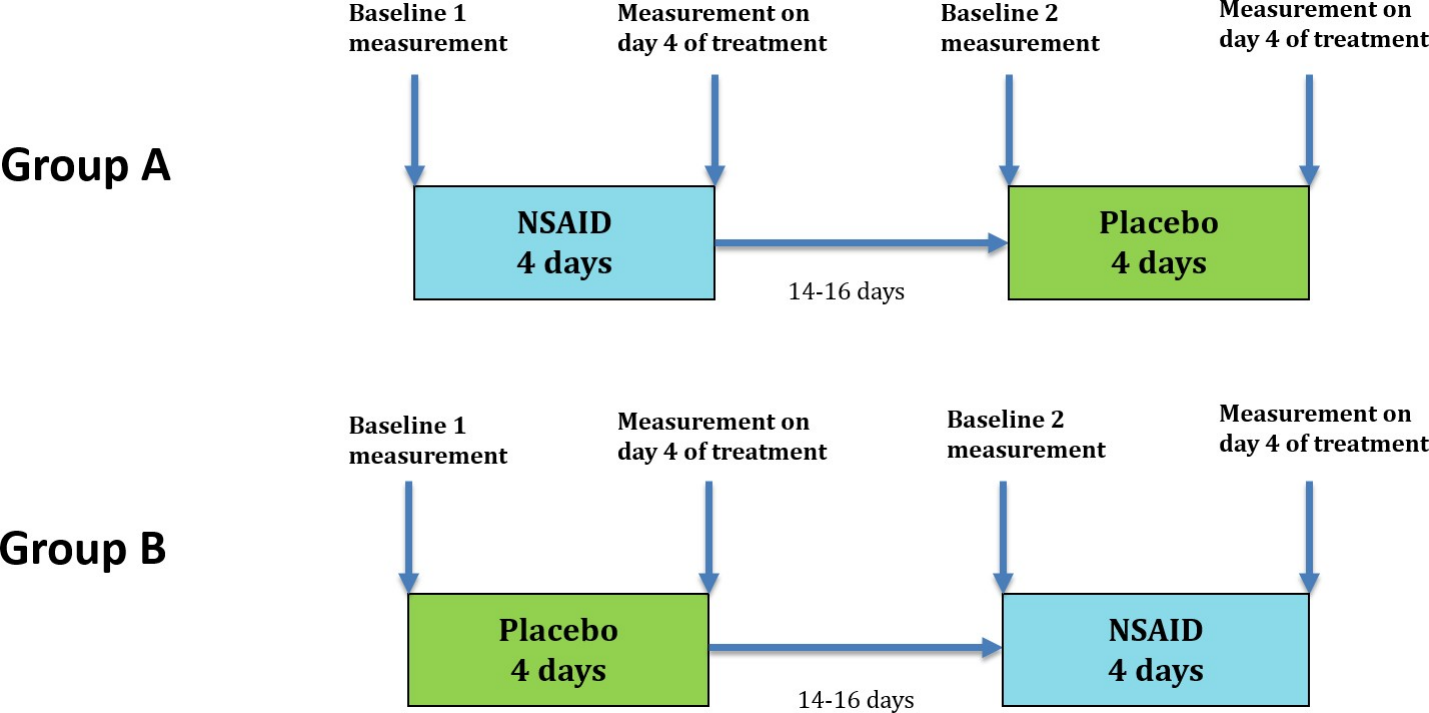
Study design.

In a crossover design, each horse was treated with meloxicam or placebo for four days respectively followed by a 14–16 day washout period between subsequent treatments (Fig 1). The order of the two treatments was randomly assigned to each horse. The owners were blinded to the order in which their horse received each treatment and were instructed to continue training according to their normal regime.

Objective movement symmetry data was collected at four time points during the study: before treatment on day 1 and after treatment on day 4 of each treatment period (Fig 1). All horses were trotted in hand on a straight line and lunged on a circle with a diameter of 10 m and 15 m (markers placed on the lunge line) in both directions. All conditions were performed twice, at two different speeds, slow (preferred speed) and fast, as judged subjectively. All conditions were also conducted on two different surfaces; a hard surface, usually consisting of packed dirt or occasionally asphalt, and a soft surface, usually an indoor arena or occasionally an outdoor arena. Surfaces thus differed between horses but were kept consistent for each individual horse throughout the study. In total, measurements were obtained under 16 different conditions for each horse at each time point. Each horse was randomly allocated to start with either of a hard/soft surface, a left/right direction and circles/straight lines. The order in which the conditions were performed throughout the study was kept consistent for each horse. In the event of technical equipment malfunctioning or uncooperative behaviour from the horse, the condition was repeated until successful data collection was completed. In the present study, only data collected from straight line in-hand trot and from the 10m lunge circles, both in preferred speed, were used.

### Drug administration, plasma sample collection and analysis

Meloxicam (Inflacam, Virbac, Kolding, Denmark) at the recommended dose of 0.6 mg/kg or placebo (vanilla yoghurt with similar colour and smell) was administered per os once daily on day 1–4 of each treatment period. The weight of each horse was assessed by means of a weight tape. Owners and riders were blinded to the order of the treatments. Treatment was administered by one of the researchers directly after the baseline movement symmetry measurement on day 1, given once daily by one of the researchers or a local stable representative (not the rider or owner) on days 2–3 and administrated by one of the researchers 1–6 hours before measurement on day 4. Plasma samples to confirm adequate meloxicam concentration were obtained by venipuncture immediately after the movement symmetry measurement on day 1 and day 4. Blood samples were collected in two lithium heparin tubes (4 ml each), plasma separated by centrifugation and stored at -80°C until analysis. Plasma samples were analysed by use of high-performance liquid chromatography with tandem mass spectrometry detection (HPLC-MS/MS, Recipharm OT Chemistry AB, Uppsala, Sweden, lower detection limit 10 ng/mL). Horses with plasma concentrations above 195 ng/mL (which is the previously published plasma concentration for reduction in lameness score [26]) after meloxicam treatment or below 20 ng/mL during any of the other sample times were included in further analysis.

### Instrumentation

The IMU system used for collection of objective motion symmetry data consisted of three sensors. One uni-axial gyroscope with a range of 300°/s was attached with a specially designed pastern wrap to the dorsal side of the right forelimb pastern. Two uni-axial accelerometers with a range of 6 g were then attached, one to the poll with a felt head bumper and one taped to the midline between the two tubera sacrale. The sensors measured 3.2 x 3.0 x 2.0 cm and had a mass of 28 g. Data was digitally recorded (8 bits) at 200 Hz and wirelessly transmitted to a handheld computer.

### Data processing

The software included in the IMU system was used for analysis of the sensor data. Recorded vertical acceleration from the head and pelvis sensor was converted to vertical displacement using a moving-window, error correcting, and double integration algorithm as described by Keegan *et al*. [14]. Stride splitting was performed based on angular sagittal plane velocity data from the limb mounted sensor’s gyroscope. Trial means of the stride-by-stride difference of the local displacement minima for the head (HDmin) or pelvic (PDmin) height between right and left limb stances were calculated. Similarly, HDmax and PDmax were calculated as the trial means of the stride-by-stride difference of the maximum head or pelvic height prior to right limb stance minus the maximum head or pelvic height prior to left limb stance. Positive values thus indicate less downward movement during stance or less upward movement produced in the push off phase (lower maximum position reached) of the right forelimb or hind limb and negative values represent asymmetries attributed to the left forelimb/hind limb.

The custom written software also aims to correct for the size of the horse by normalizing the stride-by-stride difference to the total vertical range of motion for each stride before the aforementioned trial means are calculated. Plots of HDmin and HDmax in the software output were scrutinized and outliers (up to maximum 10% of the strides) were removed.

### Statistical analysis

Data from all horses were analysed using open software (R, version 3.5.1, The R Foundation for Statistical Computing, Vienna, Austria). Mixed models were created using the lme function in the nlme package. Normality of residuals was verified using q-q plots and homoscedasticity was ensured by plotting the residuals against the fitted values. The level of significance was set to P<0.05. HDmin/HDmax are approximately twice in magnitude compared to PDmin/PDmax at the same degree of subjectively perceived lameness [29] and the thresholds for asymmetry used in this study also show this relationship. In order to avoid this resulting in selection of only forelimb asymmetry when selecting each horse’s main asymmetry as below, equal weight needed to be given to both head and pelvic parameters. Therefore, both for selection of main asymmetry and in the statistical models outlined below, head parameters were halved. The final values used were thus absolute values of HDmin/2, HDmax/2, PDmin and PDmax.

Six statistical models were implemented; three for each surface type (soft and hard). One model each for straight line data, one model each for lunge data and one model each for the 30% most asymmetric horses.

Only horses with at least one asymmetry parameter value exceeding the previously described thresholds in the ‘Baseline 1’ straight line measurement, for the surface type evaluated, were included in the models for that surface.

- Straight line models: To assess the effect of meloxicam on the horses’ main asymmetry, the asymmetry parameter (HDmin/2, HDmax/2, PDmin or PDmax) with the highest absolute value at ‘Baseline 1’ was selected for each horse. Outcome variable was the difference in absolute value before and after each treatment for this main asymmetry parameter.
- Lunge models: These two models aimed to assess the effect of meloxicam on asymmetry shown on the lunge. The direction on the circle in which each horse’s main asymmetry was highest in absolute value and indicated the same limb as on the straight line was selected. For a horse with a PDmin main asymmetry attributed to the right hind in straight line ‘Baseline 1’, the direction on the circle in which the absolute value of PDmin was greatest and attributed to right hind was chosen. Outcome variable was the difference in absolute value of the main asymmetry parameter in the selected lunge direction before/after each treatment.
- Top 30% most asymmetric horses: To assess whether meloxicam had an effect in horses with more prominent asymmetries, the horses with the top 30% highest values for their main asymmetry parameter were included in a subset of data analysed separately. Outcome variable again was the difference in absolute value before and after each treatment for the main asymmetry parameter in the selected subset in straight line trot.

In all six models, treatment was entered as fixed effect along with the difference in stride frequency before and after each treatment as a covariate. Horse was entered as a random effect (random intercept) in all models. Box plots were created for the data underlying each model illustrating the difference in main asymmetry before and after each treatment. Plots showing median values (horizontal bold line), 25th and 75th percentile (interquartile range, IQR, box limits), notches (extending 1.58*IQR/sqrt(n)), (whiskers (thin lines) extending to median +/-1.5*IQR and extreme values outside the area covered by the whiskers.

## Results

A total of 140 horses were initially screened. Out of these 32 did not present with motion asymmetries, five were excluded due to being too difficult to handle, three due to being judged as too lame (>2 degrees on a 0–5 ordinal scale) to continue their normal training and 18 due to inability to comply with the logistics of the study plan. Thus 82 horses with asymmetry values above the previously mentioned thresholds were initially selected for participation in the study. Sixteen horses were later excluded, during the study time: six due to being judged as too lame to continue their normal training, two due to being too difficult to handle and one due to planned competitions within the withdrawal time. One horse was excluded due to missing data for straight line measurements on both surfaces. Six horses were excluded due to meloxicam plasma concentrations below 195ng/mL after meloxicam treatment; or above 20ng/mL during any of the other sample times.

This resulted in inclusion of a total of 66 horses with 41 geldings and 25 mares, mean (range) age 11 years (3–22 years), height at withers 167 cm (152–180 cm), body mass 598 kg (460–750 kg). Horses included consisted of privately owned horses (n = 27), horses owned by the National Equestrian Center at Strömsholm (n = 17), by the Swedish Horse Guards Society (n = 12), and by two riding schools (n = 10). Horses were used for dressage (n = 22), show jumping (n = 21), or were all-round horses (n = 22) and one horse was used for eventing. For details of the individual horses included in the study see S1 File. The horses were not treated for lameness during six months preceding the study with the exception of two horses (treated two and four months preceding the study, respectively).

Determination of each horse’s main asymmetry, i.e. the asymmetry parameter with the highest absolute value at ‘Baseline 1’, resulted in selection of more hind limb asymmetries (72% on hard surface and 64% on soft surface) than forelimb asymmetries (Table 1). Of all the asymmetries recorded above the threshold values present in all horses, i.e. not just the main asymmetries, hind limb asymmetries were similarly found more commonly than forelimb asymmetries. For some horses different asymmetry parameters were included in each model, for example PDmin was included in the model for the soft surface and HDmin for the hard surface. Some horses showed asymmetry values above the threshold for only one surface and were thus not included in the model for the other surface. One horse was excluded from the soft surface straight line model, one other from the hard surface straight line model and one other from the soft surface lunge model due to missing data. In the end, this resulted in 57 horses in the models for the hard surface and 58 in the models for the soft surface out of the 66 horses originally included. All included horses (n = 66) remained in at least one model.

**Table 1:** Prevalence of different asymmetries in straight line trot at ‘Baseline 1’ measurement in the horses included in the study.

	Hard surface						Soft surface					
Parameter	All > threshold			Selected main asymmetry			All > threshold			Selected main asymmetry		
	n of horses	median	IQR	n of horses	median	IQR	n of horses	median	IQR	n of horses	median	IQR
HDmin	22	8.7	2.4	9	8.8	5.0	24	9.1	3.0	10	10.6	5.8
HDmax	20	8.9	2.4	7	8.9	2.3	26	8.5	3.9	11	8.6	3.4
PDmin	31	4.7	2.6	24	5.0	2.3	31	5.0	2.1	19	5.3	2.6
PDmax	32	4.6	2.1	17	5.2	2.0	31	4.6	2.1	18	5.4	3.4
Total				57	** **					58		

Distribution of the selected main asymmetries and the total number of horses with asymmetries above the thresholds (for the head absolute value >6 mm and pelvis absolute value >3 mm) for each asymmetry parameter in straight line trot. The median and IQR (interquartile range) of the absolute values for the asymmetries are also shown, head parameters are original values and not divided by two as in the statistical models.

Minor deviations from the standard protocol included the following: Differences in order of conditions between measurement time points (n = 6), increased or slightly decreased washout period (n = 13), placebo treatment increased by one day (n = 5).

**Fig 2:**
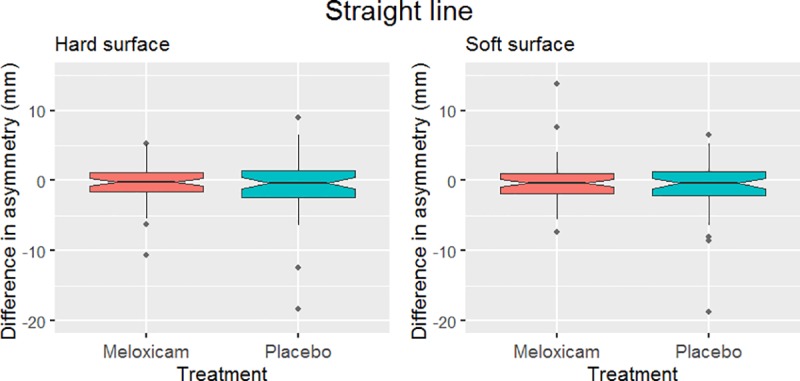
Effect of treatment on the main asymmetry parameter of the horses in straight line trot. Shown here is the difference in the main asymmetry parameter between pre-treatment on day 1 and post treatment on day 4 of each treatment (meloxicam and placebo) across 57 horses on hard surface and 58 horses on soft surface. Negative values represent a reduction in asymmetry during the treatment period. No significant effect of treatment was found (p = 0.31 for hard surface and p = 0.48 for soft surface).

**Fig 3:**
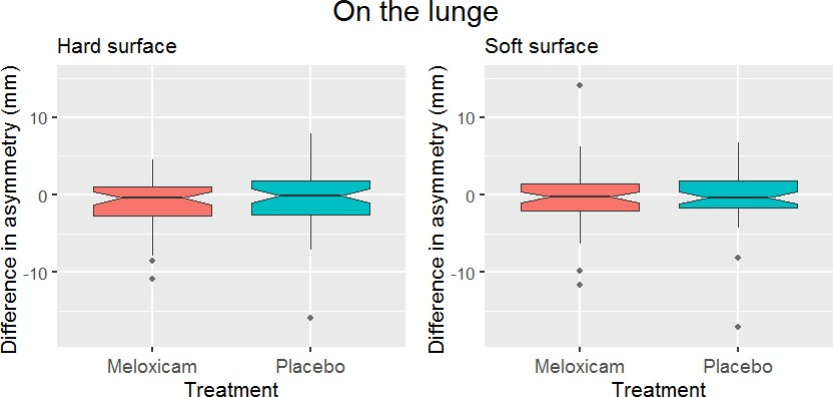
Effect of treatment on the main asymmetry parameter of the horses on the lunge. Shown here is the difference in the main asymmetry between pre-treatment on day 1 and post treatment on day 4 of each treatment (meloxicam and placebo) in the direction on the lunge where the main asymmetry parameter of each horse was most evident at ‘Baseline 1’. Negative values represent a reduction in asymmetry during the treatment period with 57 included horses on hard surface and 58 included horses on soft surface. No significant effect of treatment was found (p = 0.63 for soft surface and p = 0.75 for hard surface).

**Fig 4:**
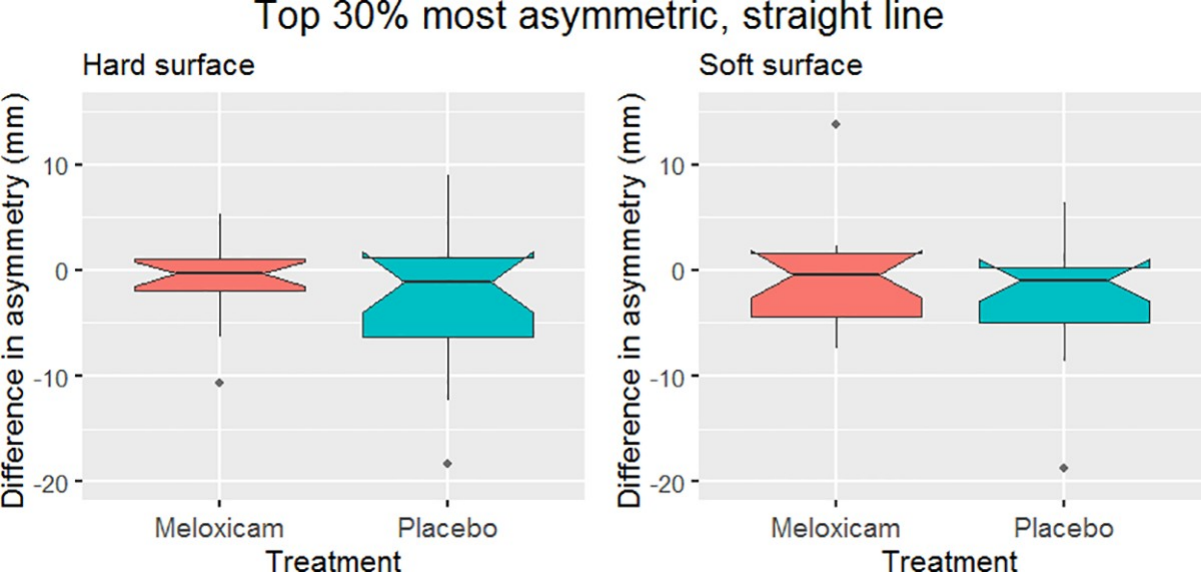
Effect of treatment in the top 30% most asymmetric horses in straight line trot. Shown here is the difference between pre-treatment on day 1 and post treatment on day 4 of each treatment (meloxicam and placebo) in the main asymmetry parameter in the top 30% horses (n = 17) with the highest degree of asymmetry. Negative values represent a reduction in asymmetry during the treatment period. No significant effect between treatments was found (p = 0.75 for soft surface and p = 0.50 for hard surface).

The effect of treatment with meloxicam was not statistically significant in any of the six mixed models created. Neither the main asymmetry on the straight (Fig 2) nor on the lunge in the direction where this asymmetry had been most evident at baseline (Fig 3) were significantly affected. There was also no significant effect of treatment found for the top 30% most asymmetric horses in straight line trot (Table 2 and Fig 4).

**Table 2:** Prevalence of different asymmetries in straight line trot in the highly asymmetric subset at ‘Baseline 1’.

	Hard surface			Soft surface		
	Selected main asymmetry			Selected main asymmetry		
	n of horses	median	IQR	n of horses	median	IQR
HDmin	3	15.2	8.7	3	14.6	1.6
HDmax	1	12.4	0	1	17.3	0
PDmin	8	7.8	2.1	7	7.7	0.8
PDmax	5	6.7	0.9	6	9.0	2.2
Total	17	** **		17		

Distribution of horses across the selected main asymmetries in straight line trot for the subset (30%) of horses showing the highest degree of asymmetry. The median and IQR (interquartile range) of the absolute values for the asymmetries are also shown. Head parameters are original values and not divided by two as in the statistical models.

The difference in stride rate significantly affected the difference in asymmetry in the model that investigated the movement asymmetry on the lunge on the soft surface. The model estimate showed that a decrease of 0.01 strides/s in stride rate led to a 0.2868 mm decrease in asymmetry (p = 0.007). The mean (SD) difference in stride rate on the lunge on soft surface was -0.01 strides/s (0.03), i.e. a small decrease over the 4 day treatment period averaged over both treatments. For values of stride frequency before and after each treatment see Table 3.

**Table 3:** Stride frequency.

	**Hard surface**			
	**Straight**		**Circle**	
	Baseline	Post treatment	Baseline	Post treatment
**Meloxicam**	1.37 (0.10)	1.35 (0.11)	1.29 (0.08)	1.27 (0.10)
**Placebo**	1.35 (0.12)	1.36 (0.11)	1.29 (0.10)	1.29 (0.10)
	**Soft surface**			
	**Straight**		**Circle**	
	Baseline	Post treatment	Baseline	Post treatment
**Meloxicam**	1.35 (0.11)	1.35 (0.12)	1.26 (0.11)	1.25 (0.10)
**Placebo**	1.35 (0.11)	1.37 (0.13)	1.26 (0.07)	1.26 (0.10)

Median (interquartile range) for stride frequency in strides/sec on the straight and circle before and after each treatment.

**Fig 5:**
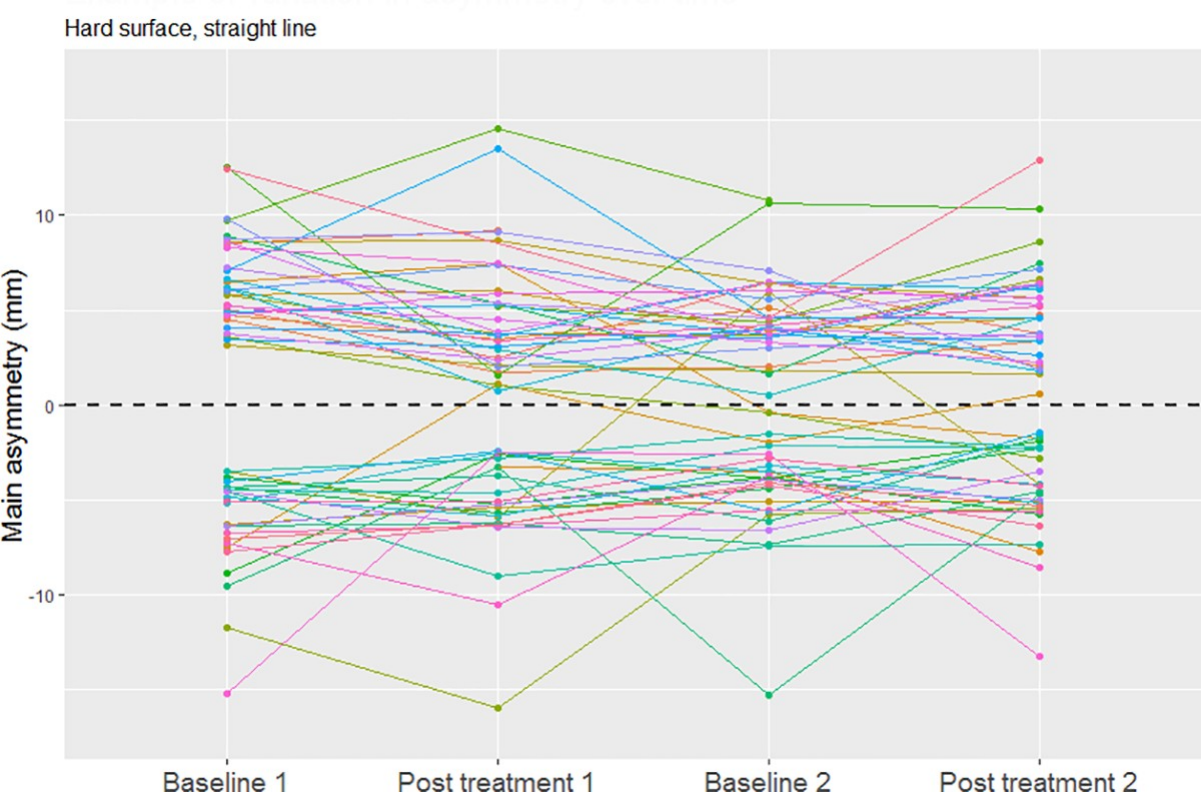
Example of variation in asymmetry over time. Change in each horse’s main asymmetry parameter across measurement time points on a hard surface, straight line. Original values are shown here (signed negative for left and positive for right sided asymmetries). Head parameters are not divided by two. For better visualisation, n = 4 outliers have been removed (outside of graph boundaries).

A considerable variation over time for the selected main asymmetry parameters was evident in some horses while others showed less variation (Fig 5). The mean absolute difference between consecutive time points across all horses for straight line assessments was median (interquartile range) 3.7 mm (6.5 mm) for head asymmetry parameters and 1.4 mm (1.9 mm) for pelvic asymmetry parameters respectively on hard surface and 3.8 mm (5.2 mm) for head and 1.6 mm (2.0 mm) for pelvic parameters respectively on soft surface (based on original values, i.e. head parameters not divided by two).

## Discussion

In this study, we have assessed the effect of treatment with meloxicam on movement asymmetries in warmblood riding horses in full training, considered free from lameness by their owners, and not recently treated for lameness. Interestingly, no significant effect of treatment on movement asymmetry was found in any of the six mixed models. This suggests that either the movement asymmetries in these horses are not generally caused by pain, but rather derives from biological variation, such as laterality or conformation, or treatment with meloxicam did not effectively moderate existing pain.

Meloxicam is commonly used in equine practice for the treatment of lame horses with orthopaedic disorders or for analgesic testing. It is a selective COX-2 inhibitor [23] and is expected to be effective in alleviating pain of inflammatory origin via reduction of prostaglandin synthesis. The non-responsiveness of the horses in the present study thus makes pain of acute inflammatory origin a less likely cause of the movement asymmetries. However, it does not exclude other types of pain being present such as chronic or neuropathic pain. In addition meloxicam has also been shown to be ineffective in reducing pain in a hoof pressure lameness model [27], which would be expected to be a predominantly nociceptive (mechanical) pain. Keegan et al. [30] evaluated the effect of phenylbutazone alone or in combination with flunixin meglumine on objectively measured lameness in a heterogeneous sample of horses with naturally occurring chronic lameness (of at least 4 months duration). On a group level, no effect was detected for phenylbutazone alone; however phenylbutazone in combination with flunixin meglumine resulted in decreased movement asymmetry. This suggests that pain in chronic lameness, non-responsive to one single class of NSAID, may be alleviated by combination treatment. Thus, treatment with only meloxicam, as in the present study, may not be potent enough to obtain a group level effect in chronic lameness cases.

In the present study, four movement asymmetry parameters for the vertical excursion of the head and pelvis were quantified to evaluate the effect of treatment. These parameters are associated with ground reaction force asymmetries [31–32] and are sensitive indicators of lameness in horses [33–37]. Validated thresholds for the discrimination between symmetric and asymmetric movement under different conditions has yet to be developed. In our study, we used the same thresholds for straight line trot as described in a previous prevalence study [15]. They are also recommended by the system developer as thresholds for detecting clinical lameness in conjunction with a full lameness examination and are similar to published confidence intervals for repeatability of asymmetries with the IMU system used [14]. This ensures that the categorization of movement asymmetries of our study can be compared to asymmetries described in other populations, including the populations where the same IMU system is used during clinical lameness exams. The variation in movement asymmetries over time in both sound and lame horses futher discussed below [38–40] should also be taken into consideration, and more research is needed to establish guidelines for use of IMU thresholds for various purposes.

In the first two models (one for each surface type) we chose to follow the evolution of the main asymmetry parameter that was determined for each horse at the time of inclusion in the study. Since four asymmetry parameters were measured simultaneously, each horse may have shown values above the thresholds for one or multiple asymmetry parameters. Selecting the most prominent asymmetry parameter might increase the chances of finding an effect of treatment in so far that possible concurrent increases in the other three asymmetry parameters during treatment are being ignored. Selecting the asymmetry with the highest magnitude also maximises the chances of selecting a parameter that is associated with a primary lameness, if present, and not a parameter describing compensatory movements of the head or pelvis resulting from a primary lameness in one of the other limbs [16,17,35,36]. Using absolute values enables a combination of left and right sided asymmetries but conceals any changes in the side of asymmetry, i.e. changes between the left and the right limb being unloaded. However, as only a small number of horses changed in the side of their main asymmetry in this study (Fig 5), this is not likely to have resulted in a major impact on the models.

In the third and fourth model the effect of treatment on the main asymmetry measured on a circular path was tested. In analogy to the first two models, the main asymmetry parameter of each horse measured at ‘Baseline 1’ was selected, and the measurement from the circle direction where it was most evident at ‘Baseline 1’ was used in the analysis. This choice was based on the previous finding that circle induced gait adaptations previously shown [18,20,36,41] are likely to increase the asymmetry selected for analysis in one direction and decrease it in the opposite direction, thus potentially concealing any effect, if both directions were included in the response variable.

The failure to detect a significant effect of meloxicam on movement asymmetry might also be due to the study population which may have included a mixture of horses both with and without pain related movement asymmetries. We therefore created a subset containing the top 30% most asymmetric horses. With higher degrees of asymmetry, our assumption was that the probability of an underlying painful pathology being present would increase. Compared to asymmetries in clinically lame horses responsive to diagnostic analgesia [16,17], the degrees of asymmetry in these subsets of horses are similar for the forelimb related parameters and higher for the hind limb related parameters. However, in the present study, no treatment effect was found in these two models either. This finding further suggests that either meloxicam does not effectively reverse pain in these horses or the measured asymmetries are not due to pain. In the model using data from the lungeing condition on the soft surface, the difference in stride frequency significantly affected the difference in movement asymmetry. A small mean decrease in stride frequency during treatments resulted in a small mean decrease in asymmetry. Stride frequency is closely related to speed which has been reported to influence asymmetry parameters on the lunge but not on the straight line [42–43]. A decrease in asymmetry due to a decrease in stride frequency, approximating a decrease in speed, is in agreement with the results in Starke et al. [43].

The majority of soft surface measurements were obtained using indoor arenas, where surfaces were expected to be fairly consistent over time. In locations where an indoor arena was not available, outdoor paddocks with a sand surface were used. Outdoor paddocks are inherently subjected to more variability due to weather conditions. However, the location of measurement (indoor vs outdoor) was kept consistent for each horse. Some owners owned a large number of included horses but each horse was ridden and trained by different rider or riders, thus training effects were expected to differ. Inclusion bias cannot be completely excluded in the present study, as owners suspecting a problem related to the locomotor apparatus may have been more motivated to volunteer their horse for participation in the study. However, a major impact of this possible bias on outcome is unlikely as the median values of movement asymmetry and the proportion of horses with values above the threshold for each of the asymmetry parameters correspond to findings in a prevalence study of a comparable population of horses [15]. Furthermore, the effect of inclusion bias in the present study would arguably have been an increased chance of finding an effect of meloxicam.

A number of horses in this study showed considerable variation over time in the measured asymmetries. This variation occurred irrespective of treatment (Fig 5), and across a relatively short period of data collection. Similar to this study a substantial variation in movement asymmetries over time has previously been shown in sound riding horses [38] and thoroughbreds deemed fit to train by their trainers [39]. In lame horses, the variation has so far only been studied between two consecutive days in horses with forelimb lameness [40] but also showed considerable day-to-day variation. If generally true in lame horses, this phenomenon has to be considered when evaluating the response to treatment in a clinical setting and when assessing a horse for soundness, for example during a pre-purchase examination, warranting further studies in the future.

In summary, the results and subsequent issues raised above warrant further investigation into the underlying cause(s) of the movement asymmetries exceeding pre-defined threshold values measured in horses in training. One possible way forward to confirm or rule out the presence of pain might be the use of diagnostic analgesia to identify possible pain loci in the lame limb. It may also be worth investigating alternative systemic analgesic treatments regimens and advanced imaging modalities. If then, underlying painful pathologies are identified in a large proportion of horses, the presence of movement asymmetry should be regarded as a severe welfare problem. If, on the other hand, no underlying pathologies linked to pain are identified, the conclusion may be that these asymmetries are due to biological variation as motor laterality or conformational asymmetries. In order to avoid unnecessary lameness examinations or to avoid erroneously failing horses during pre-purchase examinations, this remains an important research question.

## Conclusions

Treatment with four days of meloxicam did not significantly decrease the movement asymmetry measured in the horses in training included in this study. However, such a finding does not exclude that pain may be present. Therefore, the present study raises new questions with regards to whether such asymmetries are simply expressions of biological variation or related to pain/dysfunction not responsive to meloxicam treatment.

## Supporting information

S1 File Data sets used for the data analysis. Excel book containing sheets with the data sets used. Sheet ‘Full dataset’ contains data for all the 66 included horses. Sheet ‘Straight line hard surface, ‘Straight line soft surface, ‘Lunge hard surface’, ‘Lunge soft surface’, ‘Top 30% hard surface’ and ‘Top 30% soft surface’ contains the subsets and calculated extra variables used in each of the six mixed models.
(XLSX)

## References

[1] Nielsen TD, Dean RS, Robinson NJ, Massey M, Brennan ML. Survey of the UK veterinary profession: common species and conditions nominated by veterinarians in practice. Vet Rec. 2014;174(13):324–331. 10.1136/vr.101745 24570401 PMC3995283

[2] Penell JC, Egenvall A, Bonnett BN, Olson P, Pringle J. Specific causes of morbidity among Swedish horses insured for veterinary care between 1997 and 2000. Vet Rec. 2005;157(16):470–477. 10.1136/vr.157.16.470 16227382

[3] Egenvall A, Penell JC, Bonnett BN, Olson P, Pringle J. Mortality of Swedish horses with complete life insurance between 1997 and 2000: Variations with sex, age, breed and diagnosis. Vet Rec. 2006;158(12):397–406. 10.1136/vr.158.12.397 16565338

[4] Hammarberg M, Egenvall A, Pfau T, Rhodin M. Rater agreement of visual lameness assessment in horses during lungeing. Equine Vet J. 2016;48(1):78–82. 10.1111/evj.12385 25399722 PMC4964936

[5] Hewetson M, Christley TM, Hunt ID, Voute LC. Investigations of the reliability of the observational gait analysis for the assessment of lameness in horses. Vet Rec. 2006;158(25):852–858. 10.1136/vr.158.25.852 16798953

[6] Keegan KG, Wilson DA, Wilson DJ, Smith B, Gaughan EM, Pleasant RS, et al Evaluation of mild lameness in horses trotting on a treadmill by clinicians and interns or recidents and correlation of their assessments with kinematic gait analysis. Am J Vet Res. 1998;59(11):1370–1377. 9829392

[7] Keegan KG, Dent EV, Wilson DA, Janicek J, Kramer J, Lacarrubba A, et al Repeatability of subjective evaluation of lameness in horses. Equine Vet J. 2010;42(2):92–97. 10.2746/042516409X479568 20156242

[8] Parkes RSV, Weller R, Groth AM, May S, Pfau T. Evidence of the development of “domain-restricted” expertise in the recognition of asymmetric motion characteristics of hindlimb lameness in the horse. Equine Vet J. 2009;41(2):112–117. 19418737 10.2746/042516408x343000

[9] Starke SD, Oosterlinck M. Reliability of equine visual lameness classification as a function of expertise, lameness severity and rater confidence. Vet Rec. 2018 10.1136/vr.105058 30242083

[10] Serra Bragança FM, Rhodin M, van Weeren PR. On the brink of daily clinical application of objective gait analysis: What evidence do we have so far from studies using an induced lameness model? Vet J. 2018;234:11–23. 10.1016/j.tvjl.2018.01.006 29680381

[11] McCracken MJ, Kramer J, Keegan KG, Lopes M, Wilson DA, Reed SK, et al Comparison of an inertial sensor system of lameness quantification with subjective lameness evaluation. Equine Vet J. 2012;44(6):652–656. 10.1111/j.2042-3306.2012.00571.x 22563674

[12] Ishihara A, Bertone AL, Rajala-Schultz PJ. Association between subjective lameness grade and kinetic gait parameters in horses with experimentally induced forelimb lameness. Am J Vet Res. 2005;66(10):1805–1815. 16273915 10.2460/ajvr.2005.66.1805

[13] Thomsen MH, Persson AB, Jensen AT, Sørensen H, Andersen PH. Agreement between accelerometric symmetry scores and clinical lameness scores during experimentally induced transient distension of the metacarpophalangeal joint in horses. Equine Vet J. 2010;42:510–515. 10.1111/j.2042-3306.2010.00287.x 21059053

[14] Keegan KG, Kramer J, Yonezawa Y, Maki H, Pai PF, Dent EV, et al Assessment of repeatability of a wireless, inertial sensor–based lameness evaluation system for horses. Am J Vet Res. 2011;72(9):1156–1163. 10.2460/ajvr.72.9.1156 21879972

[15] Rhodin M, Egenvall A, Andersen PH, Pfau T. Head and pelvic movement asymmetries at trot in riding horses in training and perceived as free from lameness by the owner. PLoS One. 2017;12(4):e0176253 10.1371/journal.pone.0176253 28441406 PMC5404851

[16] Maliye S, Voute LC, Marshall JF. Naturally-occurring forelimb lameness in the horse results in significant compensatory load redistribution during trotting. Vet J. 2015;204(2):208–213. 10.1016/j.tvjl.2015.03.005 25862395

[17] Maliye S, Marshall JF. Objective assessment of the compensatory effect of clinical hind limb lameness in horses: 37 cases (2011–2014). JAVMA. 2016;249(8):940–944. 10.2460/javma.249.8.940 27700267

[18] Rhodin M, Roepstorff L, French A, Keegan KG, Pfau T, Egenvall A. Head and pelvic movement asymmetry during lungeing in horses with symmetrical movement on the straight. Equine Vet J. 2016;48(3):315–320. 10.1111/evj.12446 25808700 PMC5032979

[19] Buchner HHF, Svavelberg HHCM, Schamhardt HC, Barneveld A. Head and trunk movement adaptations in horses with experimentally induced fore‐or hindlimb lameness. Equine Vet J. 1996;28(1):71–76. 8565958 10.1111/j.2042-3306.1996.tb01592.x

[20] Pfau T, Jennings C, Mitchell H, Olsen E, Walker A, Egenvall A, et al Lungeing on hard and soft surfaces: Movement symmetry of trotting horses considered sound by their owners. Equine Vet J. 2015;48(1):83–89. 10.1111/evj.12374 25297461

[21] Dyson S, Greve L. Subjective Gait Assessment of 57 Sports Horses in Normal Work: A Comparison of the Response to Flexion Tests, Movement in Hand, on the Lunge, and Ridden. J Equine Vet Sci. 2016;38:1–7.

[22] Greve L, Dyson SJ. The interrelationship of lameness, saddle slip and back shape in the general sports horse population. Equine Vet J. 2014;46(6):687–694. 10.1111/evj.12222 24372949

[23] Beretta C, Garavaglia G, Cavalli M. COX-1 and COX-2 inhibition in horse blood by phenylbutazone, flunixin, carprofen and meloxicam: An in vitro analysis. Pharmacol Res. 2005;52(4):302–306. 10.1016/j.phrs.2005.04.004 15939622

[24] European Medicines Agency. Metacam. 2018 May 5 [cited 21 February 2019]. In: European Medicines Agency [Internet]. Amsterdam: European Medicines Agency—. [about 10 screens]. Available from: https://www.ema.europa.eu/en/medicines/veterinary/EPAR/metacam.

[25] de Grauw JC, van de Lest CHA, Brama PAJ, Rambags BPB, van Weeren PR. In vivo effects of meloxicam on inflammatory mediators, MMP activity and cartilage biomarkers in equine joints with acute synovitis. Equine Vet J. 2009;41(7):693–699. 19927589 10.2746/042516409x436286

[26] Toutain P, Cester CC. Pharmacokinetic-pharmacodynamic relationships and dose response to meloxicam in horses with induced arthritis in the right carpal joint. Am J Vet Res. 2004;65(11):1533–1541. 15566092 10.2460/ajvr.2004.65.1533

[27] UCVM Class of 2016, Banse H, Cribb AE. Comparative efficacy of oral meloxicam and phenylbutazone in 2 experimental pain models in the horse. Can Vet J. 2017;58(2):157–167. 28216685 PMC5234315

[28] Friton GM, Philipp H, Kleemann R. Investigation of the clinical efficacy, safety and palatability of meloxicam (Metacam) treatment in horses with musculosceletal disorders. Pferdeheilkunde. 2006;22(4):420–426.

[29] Pfau T, Sepulveda Caviedes MF, Mccarthy R, Cheetham L, Forbes B, Rhodin M. Comparison of visual lameness scores to gait asymmetry in racing Thoroughbreds during trot in-hand. Equine Vet Educ. 2018 10.1111/eve.12566

[30] Keegan KG, Messer NT, Reed SK, Wilson DA, Kramer J. Effectiveness of administration of phenylbutazone alone or concurrent administration of phenylbutazone and flunixin meglumine to alleviate lameness in horses. Am J Vet Res. 2008;69(2):167–173. 10.2460/ajvr.69.2.167 18241011

[31] Bell RP, Reed SK, Whitfield CT, Yonezawa Y, Maki H, Pai PF, et al Associations of force plate and body-mounted inertial sensor measurements for identification of hind limb lameness in horses. Am J Vet Res. 2016;77(4):337–45. 10.2460/ajvr.77.4.337 27027831

[32] Keegan KG, MacAllister CG, Wilson DA, Gedon CA, Kramer J, Yonezawa Y, et al Comparison of an inertial sensor system with a stationary force plate for evaluation of horses with bilateral forelimb lameness. Am J Vet Res. 2012;73(3):368–74. 10.2460/ajvr.73.3.368 22369528

[33] Keegan KG, Pai PF, Wilson DA, Smith BK. Signal decomposition method of evaluating head movement to measure induced forelimb lameness in horses trotting on a treadmill. Equine Vet J. 2001;33(5):446–451. 11558738 10.2746/042516401776254781

[34] Kramer J, Keegan KG, Kelmer G, Wilson DA. Objective determination of pelvic movement during hind limb lameness by use of a signal decomposition method and pelvic height differences. Am J Vet Res. 2004;65(6):741–747. 15198212 10.2460/ajvr.2004.65.741

[35] Kelmer G, Keegan KG, Kramer J, Wilson DA, Pai FP, Singh P. Computer-assisted kinematic evaluation of induced compensatory movements resembling lameness in horses trotting on a treadmill. Am J Vet Res. 2005;66(4):646–655. 15900946 10.2460/ajvr.2005.66.646

[36] Rhodin M, Pfau T, Roepstorff L, Egenvall A. Effect of lungeing on head and pelvic movement asymmetry in horses with induced lameness. Vet J. 2013;198:e39–e45. 10.1016/j.tvjl.2013.09.031 24140227

[37] Tóth F, Schumacher J, Schramme MC, Hecht S. Effect of anesthetizing individual compartments of the stifle joint in horses with experimentally induced stifle joint lameness. Am J Vet Res. 2014;75(1):19–25. 10.2460/ajvr.75.1.19 24370241

[38] Hardeman AM, Roepstorff L, Swagemakers JH, van Weeren PR, Serra Bragança FM. Variation in gait parameters used for objective lameness assessment in sound horses at the trot on the straight line and the lunge. Equine Vet J. 2019 10.1111/evj.13075 30648286 PMC6850282

[39] Sepulveda Caviedes MF, Forbes BS, Pfau T. Repeatability of gait analysis measurements in Thoroughbreds in training. Equine Vet J. 2018;50(4):513–518. 10.1111/evj.12802 29284186

[40] Rungsri PK, Staecker W, Leelamankong P, Estrada RJ, Schulze T, Lischer CJ. Use of body-mounted inertial sensors to objectively evaluate the response to perineural analgesia of the distal Limb and intra-articular analgesia of the distal interphalangeal joint in horses with forelimb Lameness. J Equine Vet Sci. 2014;34(8):972–977.

[41] Starke SD, Willems E, May SA, Pfau T. Vertical head and trunk movement adaptations of sound horses trotting in a circle on a hard surface. Vet J. 2012;193(1):73–80. 10.1016/j.tvjl.2011.10.019 22104508

[42] Pfau T, Stubbs NC, Kaiser LAJ, Brown LEA, Clayton HM. Effect of trotting speed and circle radius on movement symmetry in horses during lunging on a soft surface. Am J Vet Res. 2012;73(12):1890–1899. 10.2460/ajvr.73.12.1890 23176414

[43] Starke SD, Raistrick KJ, May SA, Pfau T. The effect of trotting speed on the evaluation of subtle lameness in horses. Vet J. 2013;197(2):245–252. 10.1016/j.tvjl.2013.03.006 23611486

